# Neurogenetic Disorders with Hearing Loss: Mechanisms, Classifications, and Emerging Insights

**DOI:** 10.1007/s11910-025-01466-y

**Published:** 2025-11-05

**Authors:** Daniel Owrang, Barbara Vona

**Affiliations:** 1 Institute for Auditory Neuroscience and InnerEarLab, University Medical Center Göttingen, Göttingen, Germany; 2 Auditory Neuroscience and Optogenetics Laboratory, German Primate Center, Göttingen, Germany; 3 Collaborative Research Center 1690 (CRC1690), University of Göttingen, Göttingen, Germany; 4 Department of Obstetrics and Gynecology, Brigham and Women’s Hospital, Harvard Medical School, Boston, MA USA; 5 Program in Medical and Population Genetics, Broad Institute of MIT and Harvard, Cambridge, MA USA

**Keywords:** Auditory genetics, Mechanism-based nosologies, Neurodevelopmental disorders, Neurodevelopmental genetics, Syndromic hearing loss

## Abstract

**Purpose of Review:**

Neurogenetic disorders associated with hearing loss represent a rapidly expanding field, with recent gene discoveries revealing convergent mechanistic themes affecting both the nervous and auditory systems. Collectively, these findings highlight shared vulnerabilities of neural and auditory tissues. We summarize gene discoveries from 2021 to 2025, moving beyond classic syndromes to highlight newly implicated genes within mechanistic categories and discuss their implications for diagnosis, counseling, and therapeutic development.

**Recent Findings:**

We describe 38 genes with combined neurodevelopmental and auditory phenotypes, providing an updated view of the field. We explore common developmental pathways and, when possible, propose explanations for the variable expression of hearing impairment observed across disorders.

**Summary:**

A deeper understanding of the mechanisms linking the nervous and auditory systems is essential for clarifying the pathogenesis of auditory syndromes. The emerging picture underscores that hearing loss can serve as an early marker of systemic neurogenetic disease that may offer a window of opportunity for timely intervention.

## Introduction

Hearing loss is among the most common sensory impairments worldwide, affecting approximately 466 million people [1]. While environmental and age-related factors account for many cases, at the younger end of the lifespan, it is now acknowledged that over half of congenital and early-onset hearing loss is attributable to genetic causes [2, 3]. Syndromic cases account for approximately 30% of congenital hearing loss [4]. Among these, a significant proportion occur in the context of neurogenetic disorders, where pathogenic variants impact both the auditory and central or peripheral nervous systems.

While traditional frameworks have categorized hearing loss as either syndromic or non-syndromic, recent advances in genomic medicine have blurred this boundary [3, 5, 6]. High-throughput sequencing, global data sharing, and the refinement of deep phenotyping have led to the discovery of many genes that link auditory dysfunction with complex neurological phenotypes. For instance, a study enrolling 100 consecutively referred children with profound-severe sensorineural hearing loss (SNHL) reported neurodevelopmental disabilities in 48% of children, including motor and cognitive disabilities [7]. These findings reveal how closely the development and function of the auditory system are intertwined with those of the central and peripheral nervous systems [8].

This overlap is not incidental. The cochlea, auditory nerve, and central auditory pathways arise from embryonic tissues with shared neural and glial lineages [9, 10]. As a result, many genes essential for cochlear homeostasis, synaptic integrity, or neuronal excitability are also expressed in brain regions, motor pathways, or cranial nerves. Moreover, the auditory pathway encompasses both peripheral components (such as the cochlea and auditory nerve) and central processing centers (in the brainstem, thalamus, and auditory cortex) [11]. The intricate development and maintenance of these structures require the coordinated action of hundreds of proteins. Disruption of such genes can, therefore, manifest as variable combinations of sensorineural hearing loss, auditory dysfunction, and broader neurological involvement.

Despite a high prevalence, hearing loss is often under-recognized as a component of various neurologic and neurodevelopmental syndromes, especially when these manifest to a severe degree in children with behavioural or attention disorders who may already be unresponsive or challenging to show appropriate responses to communication or environmental stimuli. This means that non-behavioural audiometry approaches must be used to measure hearing thresholds, and close interdisciplinary collaboration should include otolaryngologists and audiologists.

In many cases, hearing loss is among the earliest and most accessible clinical signs of an underlying neurogenetic disorder, particularly in pediatric populations where early intervention is critical [5]. Therefore, some studies have proposed that deafness may be a possible risk indicator for additional clinical features [12], underscoring the importance of collaborative cross-disciplinary medical care.

In this review, we discuss neurogenetic conditions with hearing loss reported within the past four years, focusing on genes that were only recently linked to human disease or those with recent expansions and refined views of the full phenotypic spectrum. We present a curated functional classification of these emerging gene discoveries, highlighting their clinical presentations, pathophysiologic mechanisms, and implications for diagnosis and care. Rather than revisiting well-established syndromes, we center our discussion on novel and underrecognized disorders that exemplify how auditory phenotypes can illuminate broader neurological processes. Through this perspective, we aim to reframe hearing loss not merely as a sensory symptom but as a diagnostic and mechanistic window into neurologic disease, which is a concept with growing relevance in the age of genomic medicine.

## The Auditory System: A Neurogenetic Perspective

The auditory system is a highly specialized, integrative neural network responsible for the perception of sound. Its anatomical and functional complexity spans both the peripheral and central nervous systems. Hearing is a neurobiological process: sound waves are transduced into neural signals by cochlear hair cells and transmitted via the auditory nerve to brainstem nuclei, then relayed through the midbrain and thalamus to the primary auditory cortex. The integrity of this system relies on a tightly regulated sequence of developmental events and molecular interactions, many of which are governed by genes shared with the central and peripheral nervous systems.

In recent years, the intersection of neurogenetics and auditory science has entered a transformative era. The classical division between “syndromic” and “non-syndromic” hearing loss has proven increasingly insufficient considering new discoveries from exome and genome sequencing. Since 2021, dozens of previously uncharacterized genes have been linked to disorders featuring both neurologic dysfunction and SNHL. These emerging conditions often present with developmental delay, hypotonia, epilepsy, or ataxia, but converge on molecular pathways essential to both cochlear and neural integrity. As a result, the current state of the art in this field is moving away from organ-centric phenotype definitions toward mechanism-based nosology, such as synaptopathies, axonopathies, channelopathies, and larger mechanistic frameworks, including ribosome biogenesis, mitochondrial translation, RNA processing, and ion homeostasis. This shift not only facilitates a clearer understanding of disease biology but also supports more precise approaches to diagnostic and therapeutic development.

### Recent Discoveries in Neurogenetic Disorders with Hearing Loss

**Fig. 1:**
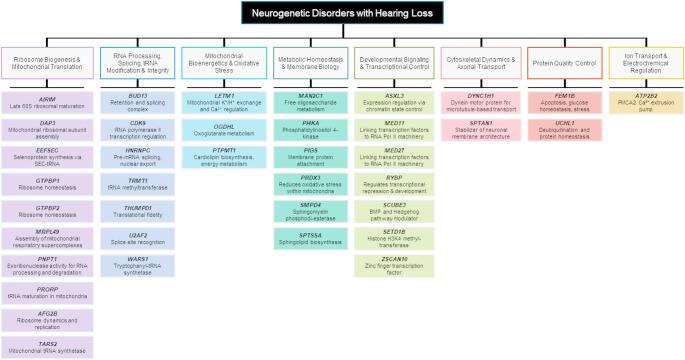
Neurogenetic disorders with hearing loss according to major mechanistic category

The next sections aim to highlight new neurogenetic disorders discovered between 2021 and 2025 that include hearing loss, as shown in Table 1; Fig. 1. We also include genes that were discovered prior to 2021 with a newly refined clinical understanding of variable hearing loss in a growing subset of patients.

**Table 1:** Genes associated with neurogenetic disorders including hearing loss described between 2021–2025

Gene	Gene name	Inheritance	Primary Mechanism	Functional Role	Phenotype summary	Secondary pathways	Ref	Year
Ribosome Biogenesis and Mitochondrial Translation								
*AIRIM*	AFG2 interacting ribosome maturation factor	AR	Ribosome maturation	Late 60S ribosomal maturation	Developmental delay with variable microcephaly and sensorineural hearing loss	mTOR	[13]	2025
*DAP3*	Death associated protein 3	AR	Mitochondrial translation	Mitochondrial ribosomal subunit assembly	Perrault syndrome spectrum	Apoptosis	[14]	2025
*EEFSEC*	Eukaryotic elongation factor, selenocysteine-tRNA specific	AR	Translation elongation	Selenoprotein synthesis via SEC-tRNA	Sensorineural hearing loss and neurodevelopmental features	Redox homeostasis	[15]	2025
*GTPBP1*	GTP binding protein 1	AR	Translational GTPase	Ribosome homeostasis	Neurodevelopmental disorder, epilepsy, microcephaly, brain atrophy, ectodermal dysplasia, spasticity, craniofacial features, sensorineural hearing loss, and vision loss	-	[16]	2024
GTPBP2*	GTP binding protein 2	AR	Translational GTPase	Ribosome homeostasis	Neurodevelopmental disorder, epilepsy, microcephaly, brain atrophy, ectodermal dysplasia, spasticity, craniofacial features, sensorineural hearing loss, and vision loss	Wnt/β-catenin signaling	[16]	2024
*MRPL49*	Mitochondrial ribosomal protein L49	AR	Mitochondrial translation	Assembly of mitochondrial respiratory supercomplexes	Sensorineural hearing loss, primary ovarian insufficiency, leukodystrophy, retinopathy, and impaired intellectual development	-	[17]	2025
PNPT1*	Polyribonucleotide nucleotidyltransferase 1	AD	RNA maturation	3´-to-5´ exoribonuclease activity for RNA processing and degradation	Spinocerebellar ataxia 25 with hearing loss	-	[18]	2022
*PRORP*	Protein only RNase P catalytic subunit	AR	Mitochondrial RNA processing	tRNA maturation in mitochondria	Perrault syndrome spectrum	-	[19]	2021
*SPATA5L1 (AFG2B)*	AAA ATPase AFG2B	AR	Ribosome assembly	Part of the 55LCC complex involved in ribosome dynamics and replication	Developmental delay, microcephaly, spastic-dystonic cerebral palsy, epilepsy, and hearing loss or non-syndromic hearing loss (DFNB119)	Ribosome recycling, genome stability and replication	[20–22]	2021
TARS2*	Threonyl-tRNA synthetase 2, mitochondrial	AR	Mitochondrial translation, aminoacylation	Mitochondrial tRNA synthetase, responsible for tRNA charging	Developmental delay, psychomotor regression, behavioural abnormalities, muscular hypotonia, cerebellar signs, epilepsy, and hearing loss	-	[23, 24]	2022
RNA Processing, Splicing, tRNA Modification and Integrity								
*BUD13*	BUD13 spliceosome associated protein	AR	Splicing regulation	Component of retention and splicing complex	Developmental delay syndrome with typical facial appearance, corneal clouding, achalasia, and progressive hearing loss	mRNA metabolism	[25]	2022
*CDK9*	Cyclin dependent kinase 9	AR	Transcription regulation	RNA polymerase II transcription regulation	CHARGE-like syndrome (Coloboma, Heart defects, Atresia choanae, Retardation of growth and development, Genital abnormalities, and Ear abnormalities and deafness)	DNA damage response	[26]	2021
*HNRNPC*	Heterogeneous nuclear ribonucleoprotein C	*De novo*	RNA splicing regulation	Pre-mRNA splicing, nuclear export	Developmental delay, behavioural abnormalities, subtle facial dysmorphology, and hearing loss	RNA stability	[27]	2023
*TRMT1*	tRNA methyltransferase 1	AR	tRNA modification	tRNA methyltransferase	Developmental delay, accompanied by variable behavioral abnormalities, epilepsy, facial dysmorphism, and hearing loss	P53 signaling	[28]	2025
*THUMPD1*	THUMP domain 1 NAT10 acetyltransferase adaptor	AR	Regulation of tRNA N4-ace- tylcytidine modification	Translational fidelity	Developmental delay, behavioural abnormalities, facial dysmorphism, and hearing loss	-	[29]	2022
*U2AF2*	U2 small nuclear RNA auxiliary factor 2	*De novo*	Spliceosome function	Splice site recognition	Developmental delay, motor and speech delay, hypotonia, seizures, behavioural abnormalities, brain malformation, facial dysmorphism, and hearing loss	-	[30]	2024
WARS1*	Tryptophanyl-tRNA synthetase 1	AR	tRNA synthetase	Tryptophanyl-tRNA synthetase (cytoplasmic)	Developmental delay, hearing impairment, microcephaly, abnormalities of the brain, skeletal system, movement/gait, and abnormal behaviour	tRNA integrity	[31]	2022
Mitochondrial Bioenergetics and Oxidative Stress								
*LETM1*	Leucine zipper and EF-hand containing transmembrane protein 1	AR	Mitochondrial ion homeostasis	Mitochondrial K⁺/H⁺ exchange and Ca^2+^ regulation	Developmental delay, optic atrophy, sensorineural hearing loss, cerebellar ataxia, epilepsy, spasticity, myopathy, bilateral cataracts, cardiomyopathy, and diabetes	Mitochondrial metabolism	[32]	2022
*OGDHL*	Oxoglutarate dehydrogenase L	AR	TCA cycle enzyme	Oxoglutarate metabolism	Neurodevelopmental spectrum disorder featuring epilepsy, hearing loss, visual impairment, and ataxia	-	[33]	2021
*PTPMT1*	Protein tyrosine phosphatase mitochondrial 1	AR	Mitochondrial bioenergetics	Cardiolipin biosynthesis, energy metabolism	Developmental delay, microcephaly, facial dysmorphism, epilepsy, spasticity, cerebellar ataxia and nystagmus, optic atrophy, bulbar dysfunction, and sensorineural hearing loss	-	[34]	2025
Metabolic Homeostasis and Membrane Biology								
*MAN2C1*	Mannosidase alpha class 2 C member 1	AR	Deglycosylation disorder	Free oligosaccharide metabolism	Developmental delay, dysmorphic facial features, severe brain anomalies including polymicrogyria, interhemispheric cysts, hypothalamic hamartoma, callosal anomalies, and hypoplasia of brainstem and cerebellar vermis, and hearing loss	-	[35]	2022
*PI4KA*	Phosphatidylinositol 4-kinase alpha	AR	Lipid kinase	Phosphatidylinositol 4-kinase	Global developmental delay, developmental encephalopathy with hypomyelinating leukodystrophy/delayed myelination and structural brain anomalies, hypotonia, spasticity, and hearing loss	Membrane identity	[36]	2021
PIGS*	Phosphatidylinositol glycan anchor biosynthesis class S	AR	GPI anchor biosynthesis	Membrane protein attachment	Global developmental delay, seizures, hypotonia, weakness, ataxia, dysmorphic facial features, multiple joint contractures, vision impairment, and hearing loss	-	[37]	2021
PRDX3*	Peroxiredoxin 3	AR	Oxidative stress/Redox regulation	Reduces oxidative stress by controlling peroxides within mitochondria	Neurodevelopmental disorder with hypotonia and hearing impairment	-	[38]	2023
SMPD4*	Sphingomyelin phosphodiesterase 4	AR	Lysosomal lipid degradation	Sphingomyelin phosphodiesterase	Neurodevelopmental delay, congenital and progressive microcephaly, epilepsy, diabetes, microcephaly, congenital arthrogryposis, early demise, retinal dystrophy, and hearing loss	Endoplasmic reticulum stress	[39]	2023
*SPTSSA*	Serine palmitoyltransferase small subunit A	*De novo*/AR	Membrane lipid metabolism	Sphingolipid biosynthesis, serine palmitoyltransferase	Developmental delay, progressive hereditary spastic paraplegia, progressive motor impairment, spasticity, variable language/cognitive impact, and hearing impairment	Axonal maintenance	[40]	2023
Developmental Signaling and Transcriptional Control								
ASXL3*	ASXL transcriptional regulator 3	*De novo*	Chromatin modification	Regulates gene expression through chromatin state control	Developmental delay, dysmorphic facial features, hypotonia, feeding difficulties, and mild conductive hearing loss	-	[41]	2021
*MED11*	Mediator complex subunit 11	AR	Transcriptional regulation (Mediator complex)	Mediator subunit linking transcription factors to RNA Pol II machinery	Global developmental delay, congenital microcephaly, seizures, movement disorder, neurodegeneration, and hearing loss	-	[42]	
MED27*	Mediator complex subunit 27	AR	Transcriptional regulation (Mediator complex)	Mediator subunit linking transcription factors to RNA Pol II machinery	Global developmental delay, bilateral cataracts, hypotonia, microcephaly, ataxia, dystonia, epilepsy, limb spasticity, facial dysmorphism, and hearing loss	-	[43]	2023
*RYBP*	RING1 and YY1 binding protein	*De novo*	Chromatin remodeling	Regulates Polycomb-mediated transcriptional repression and developmental gene programs	Developmental delay, short stature, dysmorphic facial features, hypotonia, cardiac anomalies, and conductive hearing loss	-	[44]	2025
*SCUBE3*	Signal peptide, CUB domain and EGF like domain containing 3	AR	Extracellular signaling protein	BMP and Hedgehog pathway modulator	Short stature, microcephaly, dysmorphic facial features, dental anomalies, and conductive hearing loss	-	[45]	2021
SETD1B*	SET domain containing 1B, histone lysine methyltransferase	*De novo*/AD	Epigenetic regulation/histone methylation	Histone H3K4 methyltransferase, regulates transcription via epigenetic marks	Global developmental delay, motor delay, hypotonia, delayed language development, behavioural abnormalities, seizures, and hearing loss		[46]	2021
*ZSCAN10*	Zinc finger and SCAN domain containing 10	AR	Transcriptional regulation	Zinc finger transcription factor, chromatin remodeling	Developmental delay, delayed motor development, facial dysmorphism, micrognathia, outer and inner ear malformations, and hearing loss	Pluripotency network	[47]	2024
Cytoskeletal Dynamics and Axonal Transport								
DYNC1H1*	Dynein cytoplasmic 1 heavy chain 1	*De novo*	Cytoskeletal dynamics, axonal transport	Dynein motor protein for microtubule-based transport	Peripheral neuromuscular disorders, neurodevelopmental disorders, autonomic features, behavioral disorders, movement disorders, periventricular lesions, sensory neuropathy, and hearing loss	-	[48]	2025
SPTAN1*	Spectrin alpha, non-erythrocytic 1	*De novo*/AD	Cytoskeletal structure, membrane interaction	Spectrin protein, stabilizes neuronal membrane architecture	Developmental epileptic encephalopathy, developmental delay with or without seizures, pure or complex hereditary spastic paraplegia, and hearing loss	-	[49]	2023
Protein Quality Control								
FEM1B*	Fem-1 homolog B	*De novo*	E3 ubiquitin ligase adaptor	Apoptosis, glucose homeostasis, replication stress, reductive stress	Neurodevelopmental disorder featuring behavioral issues, dysplastic/rotated ears, skeletal abnormalities, and hearing loss	-	[50]	2024
UCHL1*	Ubiquitin C-terminal hydrolase L1	AD	Conversion of ubiquitin from its pro molecule into its active form	Ubiquitin carboxyl-terminal hydrolase, responsible for protein deubiquitination and protein homeostasis	Optic atrophy, mixed cerebellar and sensory ataxia with subtle hearing loss (auditory neuropathy) possible	-	[51]	2024
Ion Transport and Electrochemical Regulation								
*ATP2B2*	ATPase plasma membrane Ca2 + transporting 2	*De novo*	Calcium transport	PMCA2 Ca²⁺ extrusion pump	Neurodevelopmental features including ataxia and dystonia, developmental delay, autism, seizures, and hearing loss	Synaptic Ca²⁺ clearance	[52]	2023

*Gene was previously described with the recent link to hearing loss described by content in the table

Abbreviations: *AD* autosomal dominant, *AR* autosomal recessive

### Ribosome Biogenesis and Mitochondrial Translation

Recent discoveries have highlighted ribosome dysfunction in both cytoplasmic and mitochondrial compartments as a major contributor to neurogenetic hearing loss. While ribosomopathies have historically been associated with bone marrow failure and craniofacial syndromes, new evidence shows that defective ribosomal assembly and translation can directly impair auditory and neural tissues. For example, biallelic variants in *AIRIM*, encoding the AFG2 interacting ribosome maturation factor, have been shown to disrupt recycling of RSL24D1 (Ribosomal L24 Domain Containing 1) from cytoplasmic pre-60S ribosome subunits back to the nucleolus, resulting in a ribosomopathy characterized by microcephaly, developmental delay, and early-onset SNHL [13]. Interestingly, *AIRIM*, also called *C1orf109*, is part of the 55LCC (SPATA5-SPATA5L1-C1orf109-CINP) complex that functions in this cytoplasmic recycling. Another protein in this complex, SPATA5L1, was also recently described. *SPATA5L1*, also known as *AFG2B*, encodes the AFG2 AAA ATPase homolog B protein, a mitochondrial AAA protease involved in both protein homeostasis and ribosome production and maturation [20–22, 53]. Its dysfunction leads to either a syndromic neurodevelopmental-auditory disorder, closely resembling the syndrome seen in patients with biallelic *AIRIM* disruption [13], or non-syndromic hearing loss (DFNB119) [20].

Defects in mitochondrial translation machinery represent a critical axis of vulnerability for auditory and neurologic systems. Unlike cytoplasmic ribosomes, mitochondrial ribosomes and their associated factors are tailored to sustain oxidative phosphorylation, and thus any disruption directly jeopardizes the energy-intensive demands of cochlear hair cells, auditory neurons, and other nervous tissues. Mitochondrial ribosomal proteins such as MRPL49 (mitochondrial ribosomal protein L49) and DAP3 (death associated protein 3) have been associated with encephalopathy and progressive SNHL [14, 17]. Patients with *MRPL49* biallelic variants present consistently with an intellectual disability syndrome that includes primary ovarian insufficiency in females and hearing impairment present in 6/9 patients in the initial discovery study [17]. Patient fibroblast analysis revealed a reduction of the small mitochondrial ribosomal subunits and a more pronounced reduction of large mitochondrial subunits and measurable defects in mitochondrial oxidative phosphorylation [17]. *DAP3* encodes a subunit of the mitochondrial ribosome and plays a role in translation of mitochondrial-encoded proteins but is also involved in cell death via apoptosis by inducing mitochondrial fragmentation through Mitochondrial Rho GTPase 1 (Miro1) [54]. Patients with biallelic *DAP3* variants present with profound SNHL, primary ovarian insufficiency, and intellectual disability [14].

Cochlear hair cells and auditory neurons have a critical dependence on efficient mitochondrial protein synthesis, given their high energy and translational demands. *GTPBP1* and *GTPBP2*, encoding GTP binding proteins 1 and 2, respectively, further exemplify this theme [16]. These GTPases are involved in ribosome-associated quality control pathways that respond to ribosomal pausing during tRNA deficiency [55]. The core phenotypes of the resulting syndrome include profound neurodevelopmental delay, epilepsy, microcephaly, brain atrophy, spasticity, pathognomonic craniofacial features, and abnormal vision and/or hearing. The clinical appearance of patients with biallelic variants in *GTPBP1* and *GTPBP2* has been noted to be so strikingly similar that the syndrome caused by variants in these two genes is called GREND (GTPBP1/2-related ectodermal neurodevelopmental) syndrome [16]. Taken together, these findings expand the ribosomopathy concept into the neurosensory domain, highlighting that translational fidelity is essential for maintaining both auditory and neurologic homeostasis.

Recent discoveries have implicated novel components beyond the structural ribosomal proteins. Human mitochondrial RNase P, comprised of three protein subunits (TRMT10C, SDR5C1, and PRORP), is responsible for 5’ end processing of mitochondrial precursor tRNAs, a vital step in mitochondrial RNA maturation [19, 56]. *PRORP*, the protein-only RNase P subunit, encodes the endonuclease subunit of the mitochondrial RNase P complex. Biallelic variants in *PRORP* are associated with a broad range of phenotypes, ranging from Perrault syndrome (with intellectual disability) in female patients and isolated SNHL in a male patient, to a severe global developmental delay with hypertonia and acquired microcephaly [19]. Analysis of patient-derived fibroblasts showed accumulation of unprocessed mitochondrial transcripts and decreased steady state levels of mitochondrial-encoded proteins. PRORP localizes to the afferent and efferent synapses of the inner hair cells and efferent synapses of the outer hair cells in the post-natal day 12 mouse cochlea, indicating a possible synaptic role [19].

*EEFSEC* encodes eukaryotic elongation factor, selenocystine-tRNA specific, a specialized elongation factor that incorporates selenocysteine during translation and is essential for biosynthesis in the human body. Selenoproteins contain selenocysteine, a rare amino acid required for brain health [57]. Individuals with biallelic variants in *EEFSEC* present with early onset neurodegeneration characterized by developmental delay, spasticity, seizures, and cerebellar atrophy [15]. Hearing impairment was only reported in 2/8 individuals in whom hearing was assessed, suggesting this may be a variable clinical feature [15]. These findings emphasize that mitochondrial translational integrity relies not only on ribosomal structure but also on ancillary processing and elongation factors, broadening the scope of neurogenetic hearing loss mechanisms linked to ribosome biology.

*TARS2* encodes the mitochondrial threonyl-tRNA synthetase 2 and plays a role in aminoacylation and mitochondrial translation. Biallelic variants in *TARS2* are linked to mitochondrial myopathy, SNHL, and epilepsy, all of which are indicative of the profound impact that mitochondrial dysfunction can have on both auditory and neural tissues [23, 24]. Hearing loss was only reported in 3/25 individuals from one study [24] and in 2/5 individuals from another [23]. While these reports highlight the critical importance of accurate mitochondrial translation for the proper function of high-energy demand tissues like those in the auditory system, it remains unclear as to why only few patients report hearing impairment.

Finally, *PNPT1* encodes polyribonucleotide nucleotidyltransferase 1, a subunit of the exosome complex that is involved in 3'-to-5' exoribonuclease activity for mitochondrial RNA processing, targeting, and degradation [58]. Deleterious variants in *PNPT1* have been associated with several distinct phenotypes, including autosomal recessive non-syndromic hearing loss (DFNB70), thought to be due to milder functional deficit compared to other variants that impact tissues with high energy demand [59]. Recently, it has been associated with Spinocerebellar Ataxia Type 25, a mitochondrial disease with sensory and/or cerebellar ataxia, SNHL, and neuropathy [18]. This heterogeneous phenotypic presentation underscores the complexity of mitochondrial RNA processing and its critical role in both the auditory system and other tissues relying on efficient energy production.

### RNA Processing, Splicing, tRNA Modification and Integrity

Parallel to the rise of ribosome-related discoveries, a growing number of RNA metabolism genes have been implicated in neurogenetic disorders with hearing loss. These include genes involved in tRNA modification, pre-mRNA splicing, and post-transcriptional regulation. For instance, *TRMT1* encodes a tRNA methyltransferase responsible for catalyzing the formation of *N2,N2*-dimethylguanosine (m2,2G) in cytosolic and mitochondrial tRNAs, a modification that is essential for tRNA structural stability [60]. Variants in *TRMT1* are associated with SNHL, epilepsy, and cognitive delay, and highlight how defects in seemingly ubiquitous RNA modifications can result in selective vulnerability of cochlear and brain neurons. Interestingly, only 4/43 individuals with biallelic variants in *TRMT1* had impaired hearing that was recapitulated in a zebrafish model [28].

*THUMPD1*, encoding THUMP domain-containing protein 1, functions as an adaptor protein for secondary modification of tRNA by cytosine acetylation [61]. Biallelic variants in *THUMPD1* cause a loss of tRNA N4-acetylcytidine modification (ac4C) function and lead to global developmental delay, speech delay, intellectual disability, behavioral abnormalities, facial dysmorphism, and ophthalmological abnormalities. Hearing impairment was identified in 6/8 individuals with available hearing assessments [29].

*CDK9*, cyclin-dependent kinase 9, that regulates transcriptional elongation via RNA polymerase II, has been linked to a neurodevelopmental disorder closely resembling CHARGE-like syndrome [26]. This included the typical defects of the external ear, hearing loss and branchial defects. Treacher Collins syndrome is caused by defects of the polymerase II pathway which is regulated by CDK9, highlighting the importance of this pathway in syndromic hearing loss [26].

Haploinsufficiency of *HNRNPC*, an RNA binding protein called heterogeneous nuclear ribonucleoprotein C, affects the alternative splicing of multiple intellectual disability-associated genes, as *HNRNPC* is involved in RNA processing [27]. *HNRNPC* is crucial for neuronal morphology, migration, and cell survival. The core phenotype of patients with heterozygous variants in *HNRNPC* includes neurodevelopmental delay, brain and behavioural abnormalities, and dysmorphic facial features. Hearing impairment was reported among other phenotypes in 2/13 individuals included in the gene discovery study [27].

*U2AF2*, U2 small nuclear RNA auxiliary factor 2, encodes a pre-mRNA splicing factor that guides the early stages of splice site selection [62]. Heterozygous, mostly *de novo U2AF2* variants were identified in 46 individuals. While most had motor delay, intellectual disability, seizures, behavioural abnormalities, or vision abnormalities, all individuals had developmental and speech delay and dysmorphic facial features. Hearing impairment was reported in 7/38 individuals (18%) who had clinical assessment of hearing [30].

Biallelic variants in *BUD13*, a spliceosome associated protein, have been linked to syndromic developmental delay and hearing loss, emphasizing the pathogenic potential of spliceosome dysfunction [25]. *BUD13* encodes an RNA binding protein that is involved in splicing and causes globally increased intron retention rates and it is suggested that *BUD13* plays a role in the release of intron-containing mRNAs into the cytoplasm [25]. Five individuals identified with nucleotide substitutions in *BUD13* reported lipodystrophy with dysmorphic facial features, microcephaly, achalasia, intellectual disability, and progressive hearing loss [25].

The increasing number of aminoacylation tRNA synthetases implicated in human diseases further highlights their role in processes other than tRNA charging, such as regulation of transcription and translation, cell signaling, and immune response [31]. *WARS1* encodes a cytoplasmic tryptophanyl-tRNA synthetase, which catalyzes the aminoacylation of tRNA with tryptophan, that is essential for protein synthesis. *WARS1* has been implicated in autosomal dominant distal hereditary neuropathy and Charcot-Marie-Tooth disease [63]. Recently, biallelic variants have been implicated as causing hearing loss in a highly variable neurodevelopmental syndrome with developmental delay and intellectual disability. Interestingly, affected individuals with a homozygous *WARS1* start loss (p.Met1?) allele showed hearing impairment, while patients with variants impacting other exons did not. A *wars1* knockout zebrafish model implicated exon 1 as a critical exon for normal hearing and opened discussions about these exons in the auditory system [31].

Central and auditory neurons, among other cell types, rely on rapid and dynamic transcriptional and translational responses to sustain their function, making them vulnerable to even subtle disruptions in RNA maturation or stability. In aggregate, these new discoveries further illustrate that cochlear and neural tissues are particularly sensitive to defects in RNA splicing, processing, and tRNA integrity.

### Mitochondrial Bioenergetics and Oxidative Stress

A third and particularly well-represented mechanistic category relates to mitochondrial metabolism and oxidative stress. Like the brain, the cochlea is a metabolically intense organ, with specialized demands for ATP in hair cells, synaptic terminals, and strial marginal cells. Unsurprisingly, recent studies have identified several new mitochondrial genes whose dysfunction results in combined auditory and neurologic impairment.

Two proteins encoding inner mitochondrial membrane proteins have recently been identified and implicated in causing a neurological and auditory syndrome. PTPMT1, protein tyrosine phosphatase mitochondrial 1, a mitochondrial phosphatase involved in cardiolipin biosynthesis and energy regulation, has been linked to an autosomal recessive primary mitochondrial disorder [34]. Cardiolipin constitutes 10–15% of the total mitochondrial phospholipid and resides in the inner mitochondrial membrane where it is involved in membrane architecture, oxidative phosphorylation, and stabilization of the super complexes [64]. Six individuals from three families were reported with a complex neurological and neurodevelopmental syndrome with core features of developmental delay, microcephaly, facial dysmorphism, epilepsy, spasticity, cerebellar ataxia, and nystagmus, as well as other variable features such as optic atrophy and bulbar dysfunction. SNHL was only reported in two unrelated individuals but was unavailable from affected individuals from the third family [34]. Secondly, the leucine zipper and EF-hand containing transmembrane protein 1 is encoded by *LETM1* that functions as an inner mitochondrial membrane protein with an osmoregulatory function controlling mitochondrial volume and ion homeostasis by acting as a mitochondrial K⁺/H⁺ exchanger as well as being involved in the uptake or extrusion of Ca^2+^ [65]. *LETM1* depletion causes mitochondrial swelling, fragmentation and loss of cristae structure, whereas overexpression causes mitochondrial elongation, cristae swelling, and matrix condensation due to osmotic imbalance [66]. Eighteen individuals with biallelic variants were recently identified and characterized with respiratory chain complex deficiencies, global developmental delay, optic atrophy, SNHL (in 78% of individuals), cerebellar ataxia and epilepsy, with additional variable features [32]. Cardiolipin and *LETM1* deficiencies impair both cochlear and cerebellar neurons, although global knockout in respective animal models is embryonically lethal and will likely make follow up detailed in vivo cochlear studies challenging.

Oxoglutarate dehydrogenase L, encoded by *OGDHL*, is a rate-limiting enzyme of the Krebs cycle and involved in mitochondrial metabolism [33]. It is strongly expressed in the brain and other neuronal tissues such as the eye and spinal cord [67]. While the Drosophila only has a single *dOgdh* gene, the zebrafish has an OGDHL ortholog and was used as a genetic model for recapitulation of phenotypes and pathway effects. A surprising level of genetic compensation was uncovered via rescue experiments, serving as a caution for disease model selection while also attempting to explain the high degree of clinical heterogeneity via monogenic, complex genetic, and non-genetic hypotheses [67]. Nevertheless, the broad clinical picture that included developmental delay, SNHL and a wide range of phenotypes asserts the necessity of additional research and patient cohorts for understanding the role of *OGDHL* in human hereditary disease.

### Metabolic Homeostasis and Membrane Biology

Recent discoveries underscore the importance of lipid and carbohydrate metabolism in maintaining cochlear integrity. *SPTSSA*, encoding serine palmitoyltransferase small subunit A, catalyzes the rate-limiting step in sphingolipid biosynthesis. Sphingolipids are both essential and cytotoxic, thus their synthesis is tightly regulated [68]. Disruption of this pathway alters sphingolipid synthesis, which is essential for neuronal membrane composition and myelin stability. Two individuals with *de novo SPTSSA* variants and another with an inherited biallelic *SPTSSA* variant were identified, where individuals presented a highly variable spastic paraplegia and motor impairment syndrome with hearing loss as a variable feature in 2/3 of affected individuals [40]. An enzyme called PI4KA, another essential protein involved in membrane-lipid metabolism, catalyzes the first step of phosphoinositide metabolism to phosphatidylinositol 4,5-bisphosphate [69]. Ten affected individuals from unrelated families were identified with biallelic variants in *PI4KA* who presented a spectrum of phenotypes ranging from severe global developmental delay with hypomyelination/delayed myelination and developmental brain abnormalities to pure spastic paraplegia, with hearing loss reported in only one patient [36].

Disorders of glycan catabolism are also important, as shown by *MAN2C1*, that encodes mannosidase alpha class 2 C member 1, an enzyme that cleaves mannose residues from the cytosolic free oligosaccharides derived from N-glycans during the degradation of misfolded N-glycoproteins and has been further implicated in apoptotic signaling [70]. Six individuals, including two fetuses, with biallelic variants in *MAN2C1* reported dysmorphic facial features, congenital anomalies, variable degrees of intellectual disability, and brain anomalies. Mild hearing loss was reported in one individual [35]. The phosphatidylinositol glycan anchor biosynthesis class S protein, encoded by *PIGS* is a subunit of the glycosylphosphatidylinositol transamidase complex that catalyzes the attachment of preformed glycosylphosphatidylinositol to proteins containing a C-terminal attachment signal [71]. Severe hearing loss was reported in 2/6 individuals included in a recently published cohort, expanding the list of recognized phenotypes [37]. However, the role of *PIGS* in the inner ear is unknown.

*PRDX3*, encoding peroxiredoxin 3, belongs to a superfamily of peroxidases and inactivates H_2_O_2_ in the mitochondria. This protective antioxidant function shields cells from oxidative damage [72]. A case report following the original discovery study described an affected individual with a homozygous nonsense variant in *PRDX3* who showed global developmental delay, cerebellar atrophy, hypotonia, speech issues, dystonia, and profound hearing impairment [38].

Finally, *SMPD4*, sphingomyelin phosphodiesterase 4, encodes sphingomyelinase that hydrolyzes sphingomyelin to form phosphorylcholine and ceramide and is essential for lipid homeostasis [73]. SMPD4 is a membrane-associated protein of the endoplasmic reticulum and nuclear envelope and interacts with nuclear pore complexes. The disease mechanism, so far, suggests that *SMPD4*-related disorder causes a lack of nuclear envelope bending that is essential for inserting nuclear pore complexes in the nuclear envelope [39]. A recent study identified five individuals from three unrelated families with biallelic loss-of-function *SMPD4* variants who had insulin-dependent diabetes and a severe neurodevelopmental disorder and microcephaly, while hearing impairment was observed in 2/5 individuals [39].

Collectively, these findings suggest that both the brain and cochlea are sensitive to perturbations in metabolic homeostasis, likely due to its reliance on finely tuned membrane lipid composition, cellular metabolism, membrane integrity, and endoplasmic reticulum-mitochondrial signaling for survival.

### Developmental Signaling and Transcriptional Control

Several new gene discoveries reflect disruptions in developmental patterning, transcriptional regulation, or protein quality control that affect both auditory and central nervous system structures. *SCUBE3*, encoding the signal peptide, CUB domain and EGF like domain containing 3 protein, acts as a BMP2/BMP4 co-receptor, recruits BMP receptor complexes into raft microdomains, and augments specific interactions between BMPs and BMP type I receptors [45]. Patients with biallelic loss-of-function variants show growth retardation, microcephaly, dysmorphic facial features, variable hypotonia and intellectual disability with conductive hearing impairment reported in 4/12 individuals [45]. Hearing impairment is thought to be due to altered bone metabolism [74].

Multiple genes have been implicated in transcriptional regulation and chromatin remodeling. These include *SETD1B*, encoding SET domain containing 1B, histone lysine methyltransferase, a catalytic SET domain protein of the histone methyltransferase complex, serving to mediate methylation of H3K4 sites for epigenetic regulation of gene transcription [75]. A 36-individual cohort with heterozygous loss-of-function variants provided the first in-depth characterization of *SETD1B*-associated phenotypes [46]. Nearly all had global developmental delay, motor developmental delay, language delay, and intellectual disability. There were several less frequent features. One individual was reported to have impaired hearing, otherwise, it was not remarked for the other patients [46]. This report should motivate deeper phenotyping opportunities to include hearing testing in patients to provide replication of this feature. The precise downstream epigenetic targets remain unknown.

*RYBP*, RING1 and YY1 binding protein, encodes a core component of the non-canonical Polycomb Repressor Complex 1 [76]. RYBP is a Polycomb group protein involved in chromatin remodeling and binds directly to the transcription factor YY1 to act as a transcription repressor [76]. A cohort study identified seven individuals with heterozygous *de novo RYBP* variants. These individuals presented a syndromic neurodevelopmental disorder with multiple congenital anomalies and identifiable dysmorphic features. Conductive hearing loss was identified in 2/7 individuals and a third individual was described with middle ear anomalies [44].

ASXL transcriptional regulator 3, ASXL3, is a chromatin modifier responsible for a neurodevelopmental syndrome in which mild conductive hearing loss has been observed [41]. It was previously associated with Bainbridge-Ropers syndrome, an autosomal dominant syndrome associated with microcephaly, developmental delay, poor or absent speech, and delayed psychomotor development [77]. A large cohort of 45 patients was reviewed, where mild conductive hearing loss was identified in three individuals, linking an auditory phenotype to this gene for the first time [41].

*MED11* and *MED27*, encoding mediator complex subunit 11 and 27 proteins, respectively, act as a physical and functional bridge between DNA-binding transcription factors and transcription machinery [43]. Biallelic variants in *MED11* [42] and *MED27* [43] are part of an emerging understanding of human pathologies involving human MED complexes. A recurrent biallelic nonsense variant in *MED11* was described as causing a lethal neurodegenerative disorder [42]. Patients reported microcephaly, profound neurodevelopmental impairment, exaggerated startle response, myoclonic seizures, progressive widespread neurodegeneration, and premature death. Hearing impairment was described in all three individuals from which auditory phenotyping was available out of the seven total individuals. Protein expression was identified in the mouse and zebrafish inner ear and a zebrafish knock out model recapitulated the phenotypes seen in patients, including hearing loss [42]. Fifty-seven individuals were collected in the *MED27* discovery study wherein patients were described with a continuum of phenotypes comprising developmental delay/intellectual disability, most had cataracts, hypotonia, microcephaly, dystonia, ataxia, and epilepsy. Hearing loss was reported in a single individual [43]. Whether this remains as a feature due to other genetic or environmental causes or is an underrecognized part of this syndrome remains to be clarified; however, these two genes further highlight that defects in global transcriptional regulation can manifest with neurosensory phenotypes.

Finally, a newly implicated transcription factor in neurodevelopmental and auditory syndromes called *ZSCAN10*, zinc finger and SCAN domain containing 10, that controls pluripotency of embryonic stem cells [78]. Biallelic *ZSCAN10* loss-of-function variants were identified in seven affected individuals who consistently reported global developmental delay, facial asymmetry and malformations of the outer ear. Cerebral MRI showed dysplasia of the semicircular canals as an anatomical malformation of the inner ear, and 4/5 individuals were confirmed with SNHL [47].

### Cytoskeletal Dynamics and Axonal Transport

DYNC1H1 and SPTAN1 are integral components of cytoskeletal dynamics and axonal transport, which are critical for maintaining neuronal stability and axon functionality. The dynein cytoplasmic 1 heavy chain 1 protein, encoded by *DYNC1H1*, serves as the only motor-supported retrograde cargo transport complex subunit within neuronal axons [79]. Forty-seven patients were recruited in an effort for deeper phenotyping of the syndrome [48]. The predominant phenotype involved neuromuscular features, for example, hypotonia and deterioration (noted in several patients). Novel multisystem features such as immunodeficiency, hearing impairment and skeletal manifestations were also present [48]. SPTAN1, spectrin alpha, non-erythrocytic 1, a spectrin protein, belongs to a family of widely distributed filamentous cytoskeletal proteins. *SPTAN1* encodes a membrane scaffolding protein for maintenance of integrity of myelinated axons, axonal development and synaptogenesis [80]. Heterozygous or *de novo* variants in *SPTAN1* have been previously implicated in several syndromes with either developmental delay with or without epilepsy, spastic paraplegia or distal hereditary motor neuronopathy. Gene enrichment analysis of the 100,000 Genomes Project and gene matching constructed a large cohort of *SPTAN1* patients wherein three subgroups were defined: developmental epileptic encephalopathy, milder phenotypes of developmental delay with or without seizures, and patients with pure or complex hereditary spastic paraplegia/hereditary ataxia [49]. Two individuals were identified with hearing loss; it was described as mild in one and with adult onset in the other and remains a rare feature of this complex syndrome [49]. These genes underscore how cytoskeletal and axonal dysfunctions can disrupt neuronal signaling and sensory processing, particularly in the auditory system. As hearing loss is only a newly recognized feature due to large cohort aggregation, research into disease mechanisms in the auditory system is needed.

### Protein Quality Control

FEM1B and UCHL1 are both involved in protein quality control through distinct mechanisms but with a shared function of maintaining protein homeostasis. Fem-1 homolog B, encoded by *FEM1B*, acts as an E3 ubiquitin ligase within the ubiquitin-proteasome system, which is essential for targeted degradation of damaged or misfolded proteins. A recurrent *de novo* missense variant in *FEM1B* in five individuals was identified as causing a severe neurodevelopmental disorder with behavioral phenotypes and variable malformations, facial dysmorphism, with hearing loss present in two individuals, emphasizing its crucial role of ubiquitin-mediated proteostasis to neurosensory health [50]. The dysfunction in protein degradation processes associated with FEM1B may lead to disruption of oxidation-reduction balance, contributing to neurological phenotypes and SNHL. *UCHL1* encodes ubiquitin C-terminal hydrolase L1, a key ubiquitin carboxyl-terminal hydrolase, and plays an essential role in protein quality control by stabilizing monoubiquitin, preventing degradation and increasing the available pool of ubiquitin for tagging proteins destined for degradation by the proteasome [81]. Deleterious variants in *UCHL1* have been recently linked to optic atrophy, ataxia with subtle hearing loss (auditory neuropathy), demonstrating its importance in maintaining protein homeostasis [51]. Given its involvement in synaptic function, defects in UCHL1 could contribute to neurodegeneration and hearing loss, further highlighting the importance of protein quality control in auditory synaptophies.

### Ion Transport and Electrochemical Regulation

Another rapidly evolving area involves genes that regulate ion transport and endolymph homeostasis. These mechanisms are essential not only for cochlear transduction but also for action potential propagation and synaptic fidelity in auditory neurons. *ATP2B2* encodes the ATPase plasma membrane Ca²⁺ transporting 2 or plasma membrane Ca²⁺-ATPase type 2 (PMCA2) protein, a Ca²⁺ extrusion pump that helps maintain intracellular calcium levels in inner hair cells, and was originally linked to autosomal dominant non-syndromic hearing loss (DFNA82) in humans in 2019 [82], but identified as a deafness gene in the mouse nearly two decades prior [83]. Deleterious variants have been associated with a neurodevelopmental syndrome including hearing loss in some individuals [52]. Collectively, these findings reveal that subtle disturbances in ion handling can produce profound auditory dysfunction, particularly when affecting the stria vascularis, synaptic clefts, or auditory nerve fibers.

### Integrative Perspective: Mechanistic Convergence in Auditory and Neurologic Dysfunction

The diversity of cellular mechanisms represented in recent gene discoveries underscores the biological complexity of hearing loss in neurogenetic disorders (Table 1). Yet despite this mechanistic breadth, a unifying theme is the selective vulnerability of auditory and neural systems to disruptions in cellular homeostasis. Both cochlear hair cells and neurons rely on sustained transcriptional activity, rapid protein turnover, precise ion gradients, and uninterrupted mitochondrial function. These demands render them particularly susceptible to the cumulative stress imposed by variants in genes that regulate basic intracellular processes. Importantly, many of the disorders identified in the past four years show auditory symptoms early in the disease course, sometimes preceding overt neurologic signs. This has significant implications for clinical care: hearing loss in a child with hypotonia, developmental delay, or unexplained regression should prompt consideration of a neurogenetic diagnosis. As this list of genes continues to grow, the value of a mechanism-based diagnostic framework becomes increasingly evident, not only to classify disease more precisely, but to open pathways for therapeutic targeting, particularly in conditions where early intervention may alter developmental trajectories.

### The Challenge of Clinical Heterogeneity

Many of the genes listed in Table 1 have only recently been associated with hearing impairment, often based on variable or inconsistent reporting. In some syndromes, hearing loss may be overlooked or deprioritized when more severe features dominate the clinical picture. Reliable audiological testing in affected children can also be challenging; for example, auditory brainstem response testing may require sedation, which is sometimes avoided due to the added stress in medically complex patients.

In cases where hearing loss is a variable feature, this may reflect true biological heterogeneity. One possibility is transcript-specific vulnerability, in which deleterious variants affecting inner ear-specific isoforms cause hearing loss, while variants in other exons are tolerated by the auditory system but cause non-auditory features. This is a mechanism proposed in the variable hearing impairment observed among *WARS1* patients [31]. Alternatively, variability may result from differential organ vulnerability or from variant-specific effects (e.g., residual activity versus complete loss-of-function). In such scenarios, residual cochlear function may be sufficient to preserve normal hearing, produce only mild impairment, or lead to delayed hearing loss that is easily missed.

### Clinical Applications and Future Directions

The rapid expansion of neurogenetic genes associated with hearing loss in recent years has had a profound impact on both clinical practice and research strategy. With many of these conditions presenting first, or most conspicuously, with auditory symptoms, hearing loss now plays a critical role as a diagnostic entry point into broader neurogenetic syndromes [5]. Importantly, many of the newly identified genes do not involve cochlear-specific proteins but rather encode fundamental regulators of translation, RNA processing, or mitochondrial homeostasis. This means that mechanistic rather than phenotypic suspicion is increasingly required for diagnosis: a child with progressive SNHL and global developmental delay may have deleterious variants in a tRNA synthetase, a mitochondrial phosphatase, or a spliceosome factor, none of which would have been considered in previous gene panels limited to “hearing loss” associated genes alone.

As a result, there is growing consensus that individuals with SNHL and any neurologic symptoms, even subtle hypotonia, mild language delay, or balance concerns, should be evaluated with comprehensive genomic testing, ideally through exome or genome sequencing. Targeted panels still play a role, particularly in neonatal screening, but may miss recently described genes or gene content with atypical presentations. Importantly, functional annotation of novel variants, especially in genes with pleiotropic mechanisms, requires integration of audiologic, neurologic, and molecular data, emphasizing the need for collaboration across disciplines. Audiologists, medical geneticists, neurologists, and developmental specialists must work together not only to diagnose these disorders but also to define the full spectrum of phenotypes, including the pattern, progression, and type of hearing loss.

Advances in cochlear implant candidacy and outcomes are also being shaped by these mechanistic insights. For example, auditory synaptopathies involving *OTOF* [84] or *SLC17A8* [85], among the first to be described, have paved the way for understanding newer synaptic and ion channel disorders (e.g., *UCHL1*), in which cochlear implants may be beneficial, or predicted to be beneficial despite the few patients identified so far with *UCHL1* variants, in spite of poor performance on standard objective measures of hearing testing. Conversely, children with progressive central neurodegeneration may show limited benefit from implantable devices despite early intervention, highlighting the importance of pathway-informed prognostication. Future cochlear implant protocols may increasingly incorporate genetic etiology into candidacy and counseling.

Therapeutically, these discoveries offer a roadmap for precision medicine. Some disorders caused by defects in ribosome recycling or tRNA modification may be amenable to RNA-based therapies, such as antisense oligonucleotides or tRNA stabilization compounds, which are in development for related neurologic syndromes. Mitochondrial translation or metabolic homeostasis disorders (e.g., *PRORP*, *MRPL49*, or *PRDX3*) may benefit from early metabolic interventions, antioxidants, or emerging mitochondrial gene therapies, though these remain experimental. For a small but growing number of genes (e.g., *AIRIM*, *WARS1*) [31], functional rescue has been demonstrated in model systems, underscoring the importance of early diagnosis and preclinical pipelines.

Looking forward, progress in this field will depend on expanding the functional annotation of newly implicated genes. While sequencing has accelerated discovery, many variants remain with an interpretation of uncertain significance, particularly in undercharacterized genes with broad expression. Human cell-based models, inner ear organoids, and single-cell transcriptomics are likely to play an essential role in clarifying gene function in cochlear subtypes and guiding target validation. Integrating audiologic phenotypes into genotype-driven natural history studies will be essential, particularly for genes where hearing loss may precede or predict neurologic progression. Finally, large-scale cross-disciplinary registries will be crucial to building the evidence base for variant interpretation, outcomes for research, longitudinal tracking, and future therapeutic trials.

In summary, the recent expansion of neurogenetic disorders involving hearing loss has redefined how these conditions are approached in clinical and research settings. Hearing loss should be reframed as potentially more than simply an otologic concern, but rather a potential highly interpretable signal of deeper biological dysfunction, one that can guide early diagnosis, therapeutic selection, and mechanistic understanding across the spectrum of pediatric neurology and genetics.

## Conclusion

Recent advances in gene discovery have transformed understanding of the molecular basis of neurogenetic disorders combined with hearing loss. Diverse pathways converge to play a role in neurogenetic and auditory disorders. Chief among these is the observation that auditory and neural systems share unique metabolic and structural demands that render them especially sensitive to perturbations in fundamental cellular processes.

Clinically, these findings underscore the diagnostic value of hearing loss as both a sentinel and a core feature of multisystem neurodevelopmental syndromes. Recognition of these patterns has immediate implications for genetic testing strategies, anticipatory guidance, and early interventions. Looking forward, therapeutic approaches ranging from metabolic supplementation to targeted molecular therapies may hold promise for subsets of these conditions. Together, these discoveries expand the mechanistic frameworks into the auditory domain, highlighting the importance of cross-disciplinary research in neurology, genetics, and auditory science.

### Key References

- World Health Organization. World report on hearing [Internet]. Geneva: World Health Organization; 2021. Available from:https://apps.who.int/iris/handle/10665/339913.
  - This is a key reference in the field for understanding the epidemiology of hearing loss.
- Sutton AE, Goldman J. Syndromic Sensorineural Hearing Loss. In: StatPearls [Internet]. Treasure Island (FL): StatPearls Publishing; 2025 [cited 2025 Aug 24]. Available from:http://www.ncbi.nlm.nih.gov/books/NBK526088/.
  - This is a reference that describes the basic information about hearing and common syndromes of the auditory system.
- Vona B. Rethinking non-syndromic hearing loss and its mimics in the genomic era. Eur J Hum Genet. 2025 Mar;33(2):147–50.
  - This article discusses how advances in genomic technologies have changed the way non-syndromic hearing loss and syndromes that mimic non-syndromic hearing loss at onset are diagnosed and understood. It emphasizes that there are many syndromes that initially appear as isolated hearing loss and the importance of molecular genetic testing for understanding potential syndromes.
- Chilosi AM, Comparini A, Scusa MF, Berrettini S, Forli F, Battini R, et al. Neurodevelopmental disorders in children with severe to profound sensorineural hearing loss: a clinical study. Dev Med Child Neurol. 2010 Sept;52(9):856–62.
  - This article evaluated the frequency and type of additional developmental disabilities in children with sensorineural hearing loss and evaluated the relationship between neurodevelopmental disorders and profound sensorineural hearing loss.

## Data Availability

No datasets were generated or analysed during the current study.
